# Fanconi anemia core complex-dependent HES1 mono-ubiquitination regulates its transcriptional activity

**DOI:** 10.1186/s13104-018-3243-7

**Published:** 2018-02-20

**Authors:** Cédric S. Tremblay, Feng Fei Huang, Georges Lévesque, Madeleine Carreau

**Affiliations:** 1 Australian Centre for Blood Diseases, Monash University, Melbourne, Australia; 2 Francis Family Liver Clinic, University Health Network, Toronto, ON Canada; 3 Department of Psychiatry and Neurosciences, Université Laval, Quebec, QC Canada; 4 Department of Pediatrics, Université Laval, CHUL, 2705 Boul. Laurier, RC-9800, Quebec, QC G1V 4G2 Canada

**Keywords:** Hairy-Enhancer of Split-1, HES1, Fanconi anemia, Ubiquitination, Mono-ubiquitination

## Abstract

**Objective:**

The Hairy Enhancer of Split 1 (HES1) is a transcriptional repressor that regulates cellular proliferation and differentiation during development. We previously found an interaction between HES1 and Fanconi anemia (FA) proteins. FA is a hematological and developmental disorder caused by mutations in more than 20 different genes. Eight FA gene products form a nuclear core complex containing E3 ligase activity required for mono-ubiquitination of FANCD2 and FANCI, both of which are FA proteins. Given that HES1 interacts with members of the FA core complex, the aim of this study was to determine whether HES1 is mono-ubiquitinated via the FA core complex.

**Results:**

We show that HES1 is mono-ubiquitinated on a highly-conserved lysine residue that is located within a FA-like recognition motif. HES1 modification is dependent on a functional FA complex. Absence of HES1 mono-ubiquitination affects transcriptional repression of its own promoter. This study uncovers a novel post-translational modification of HES1 that regulates its transcriptional activity and suggests that ubiquitination of HES1 occurs in a FA core complex-dependent manner.

## Introduction

Transcriptional repressors of the Hairy-related basic helix-loop-helix (bHLH)-type family include Hairy and Enhancer of Split homologue 1 (HES1) [1–4]. We previously reported that HES1 interacts with Fanconi anemia (FA) proteins that compose the FA core complex, which modulates its transcriptional activity and self-repression [5, 6]. Given the interaction of HES1 with FA core complex components and the FA core complex E3 ligase activity [5–10], we explored the possibility that HES1 might be a target of the FA core complex E3 ligase activity. Here, we present evidence that HES1 is mono-ubiquitinated in a FA protein-dependent manner. The HES1 protein sequence displays a putative FA-like recognition motif, which contains a conserved lysine residue. Mutation of this lysine residue alters HES1 transcriptional activity. We propose that HES1 is a putative protein substrate of the FA core complex ubiquitin ligase activity, and that this post-translational modification regulates its transcriptional activity.

## Main text

### Methods

#### Cells, plasmids and antibodies

Cells used include: 293T (HEK293T; ATCC, Cedarlane Laboratories), HeLa (ATCC) COS-1 (ATCC, CRL-1650), PD430T (FA-A) fibroblast cell lines (gift) [5, 6]. Cells were grown at 37 °C, in 5% CO_2_ in DMEM media supplemented with 10% FCS. Plasmids used include: Myc-tagged ubiquitin (pCW7) or Myc-tagged ubiquitin K48R mutant (pCW8) coding vectors, HES1pro-*Luc;* pGL3-p21^*CIP1/WAF1*-2326/+16^ (p21pro-*Luc*) plasmids. Antibodies include: anti-FANCD2 (SantaCruz Biotechnologies clone FI-17 SC-20022 or Novus Biologicals NB100-182), rabbit polyclonal anti-HES1 (Chemicon AB5702 or SantaCruz Biotechnologies clone H-140, SC-25392); monoclonal anti-β-tubulin (E7, Developmental Studies Hybridoma Bank); monoclonal anti-cMyc (SantaCruz Biotechnologies clone 9E10, SC-40), monoclonal anti-HA (Roche Diagnostics, clone 12CA5, #11583816001), anti-ubiquitin (SantaCruz Biotechnologies clone N-19; SC-6085), Goat anti-mouse IgG-HRP conjugated (SantaCruz Biotechnologies, SC-2064), Goat anti-rabbit IgG-HRP (SantaCruz Biotechnologies, SC-2004). Transfections were performed using Lipofectamine 2000 (Invitrogen).

#### Immunofluorescence, immunoprecipitation and Western blotting procedures

Whole cell lysates were subjected to immunoblot or immunoprecipitation (IP) as described in [5, 6]. For IP, equal amounts of protein were incubated overnight at 4 °C with 2 µg of antibodies followed by incubation with protein-G magnetic beads (Invitrogen). IP were resolved by sodium dodecyl sulfate–polyacrylamide gel electrophoresis (SDS-PAGE) and subjected to Western blotting with antibodies as indicated in each figure. Negative IP controls were performed using either mouse or rabbit serum. For immunofluorescence, HeLa cells were fixed in paraformaldehyde (2%) and permeabilized with Triton 0.3% and labeled with primary antibodies followed with secondary antibodies as described previously in [5, 6] and in the figure legend. Randomization was performed during labeling. Labeled cells were visualized using the Nikon E800 fluorescent microscope equipped with a C1 confocal system (Nikon Canada) at 100× magnification.

#### Luciferase assays

COS-1 or FA-A fibroblast cells seeded in six-well plates were transfected with 0.38 µg of the HES1pro-*Luc* vector, or 0.75 µg of the p21pro-*Luc* (pGL3-p21^*CIP1/WAF1*-2326/+16^) promoter vectors together with 0.125 μg of the pCMVLacZ control plasmid, as previously described [6]. The total amount of plasmid DNA was equalized between transfections using empty vectors. Cells were treated or untreated with mitomycin C (120 ng/ml) for 24 h. Cell extracts were prepared 48 h following transfection and assayed for luciferase activity using the Luciferase assay system (Promega). Extracts were randomly distributed for analysis. Transfection efficiencies were normalized with pCMVLacZ using the β-galactosidase luminescence kit II (Clontech). Each experiment was performed at least twice in triplicates. Luciferase activity is expressed as relative luciferase units (RLU).

#### Statistical analyses

Data were expressed as means ± standard errors of the means (SEMs). Statistical analyses were performed using the GraphPad Prism software (version 5.0b, GraphPad Software Inc., San Diego, CA). Paired and unpaired two-tailed Student’s t tests were used to compare groups. Differences between means were evaluated using 2-way ANOVA with Bonferroni correction test. p values less than 0.05 were considered significant.

### Results

#### HES1 is mono-ubiquitinated in a FA core complex-dependent manner

**Fig. 1:**
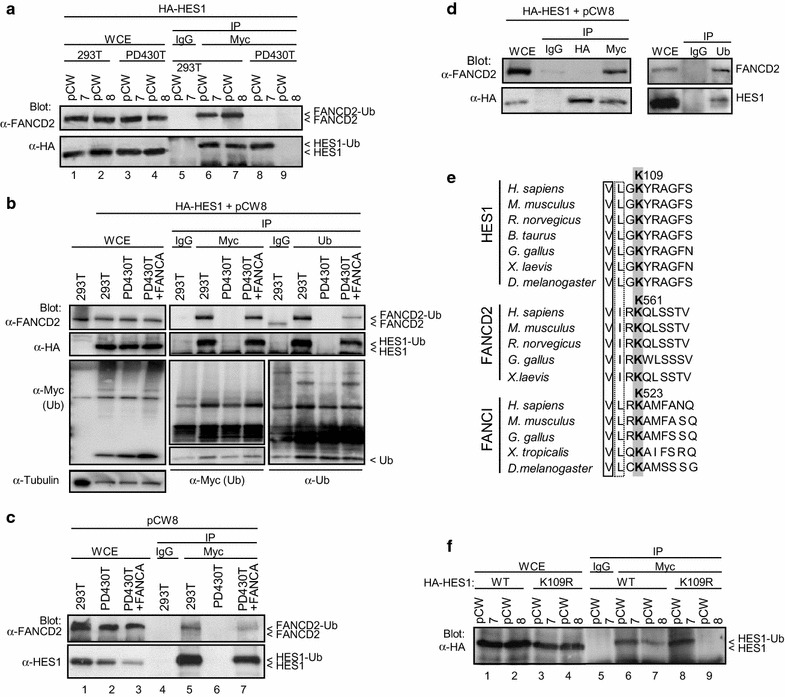
HES1 mono-ubiquitination and its dependence on a functional FA complex. **a** In vivo ubiquitination of HES1. 293T and PD430T (FA-A) cells expressing HA-HES1 and Myc-tagged ubiquitin (pCW7) or the Myc-tagged ubiquitin K48R mutant (pCW8) were subjected to immunoprecipitation using anti-Myc antibodies or control IgG and were immunoblotted against HES1 and FANCD2. **b** Complementation of FA-A cells restores HES1 ubiquitination. PD430T (FA-A) cells and PD430T complemented with FANCA were transfected with HA-HES1 and the Myc-tagged ubiquitin K48R mutant (pCW8). Cell lysates were subjected to immunoprecipitation with anti-Myc or anti-ubiquitin antibodies. Western blotting was performed with the indicated antibodies. **c** Analysis of endogenous HES1 ubiquitination in FANCA mutant fibroblasts (PD430T). PD430T cells and PD430T complemented with FANCA were transfected with the Myc-tagged ubiquitin K48R mutant (pCW8) and were subjected to immunoprecipitation with anti-Myc antibodies. Western blotting was performed with the indicated antibodies. **d** Immunoprecipitation of HA-HES1 using anti-Myc or anti-HA antibodies. 293T cells transfected with HA-HES1 and the Myc-tagged ubiquitin K48R mutant (pCW8) were subjected to immunoprecipitation with anti-HA or anti-Myc antibodies (left panel). Immunoprecipitation of endogenous proteins using anti-ubiquitin antibodies (right panel). Western blotting was performed with the indicated antibodies. **e** Alignment of the conserved region of HES1, FANCD2 and FANCI containing the putative FA recognition sequence. The peptidic sequence of HES1, FANCD2 and FANCI possess a V(I/L)XK sequence highly conserved through evolution. **f** HES1 lysine 109 is crucial for HES1 mono-ubiquitination. 293T cells were transfected with *HA*-*HES1* or *HA*-*HES1*^*K109E*^ coding vectors with the Myc-tagged ubiquitin K48R mutant (pCW8). Cell lysates were subjected to immunoprecipitation with anti-Myc antibodies or IgG control. Western blotting was performed with anti-HA antibodies. Antibody dilutions were used as follows: anti-HA at 1:5000; anti-HES1 at 1:1000; anti-FANCD2 at 1:1000; anti-tubulin at 1:10,000; anti-Myc at 1:500; anti-ubiquitin at 1:500; followed by secondary antibodies, anti-mouse, 1:10,000; anti-rabbit, 1:20,000

To determine whether HES1 as a FA complex partner is a substrate of the complex E3 ligase activity, 293T cells were transfected with Myc-ubiquitin (pCW7) or the dominant negative Myc-tagged ubiquitin mutant K48R (pCW8). The K48R mutant prevents the formation of multi-ubiquitin chains (polyubiquitination) through its lysine 48 residue, thus revealing mono-ubiquitinated forms [11]. Whole cell extracts were subjected to immunoprecipitation using anti-Myc antibodies (or mock IgG) and immunoblotted for HES1 and FANCD2 as a mono-ubiquitinated control protein. Both HES1 and FANCD2 were detected in Myc-ubiquitin immunoprecipitates from cells transfected with either wild-type ubiquitin (Fig. 1a lane 6) or the ubiquitin K48R mutant suggesting that HES1 is mono-ubiquitinated (Fig. 1a lane 7). We next examined HES1 ubiquitination in FA pathway-defective cells. HES1 was detected only in FA-A cells that expressed the wild-type ubiquitin protein but not the dominant negative ubiquitin K48R (Fig. 1a lanes 8 and 9, respectively) suggesting that mono-ubiquitination of HES1 requires a functional FA complex/pathway. Indeed, HES1 mono-ubiquitination (as well as FANCD2) was restored in FA-A cells after complementation with the *FANCA* gene as shown by immunoprecipitation using both anti-ubiquitin and anti-Myc antibodies (Fig. 1b). Immunoprecipitation using anti-Myc antibodies in FA-A mutant cells did not show any mono-ubiquitinated forms of endogenous HES1 or the long form of FANCD2 (Fig. 1c lane 6). However, endogenous HES1 mono-ubiquitination (as well as FANCD2) was restored in FA-A cells after complementation with the *FANCA* gene (Fig. 1c lane 7). Immunoprecipitation of HES1 using anti-Myc antibodies did not result from HES1 interaction with ubquitinated proteins such as FANCD2 since immunoprecipitates using anti-HA antibodies showed the presence of HES1 but not FANCD2 (Fig. 1d, left panel). Also, immunoprecipitates using anti-ubiquitin antibodies confirmed that HES1 is ubiquitinated such as FANCD2 (Fig. 1d, right panel).

We searched HES1 conserved regions and lysine residues for a putative FA recognition sequence. We found that the lysine 109 residue, which is conserved in the HES1 sequence throughout evolution, is located within a putative FA recognition motif V(L/I)XK (Fig. 1e). To determine whether the conserved lysine 109 is crucial for HES1 mono-ubiquitination, a K109E mutant form of HES1 was generated. The mono-ubiquitinated form of HES1 was immunoprecipitated only in cells expressing the wild-type HES1 but not the K109E mutant (Fig. 1f) suggesting that this residue is crucial for HES1 mono-ubiquitination. Together these results suggest that HES1 is monoubiquitinated in a FA core complex-dependent manner.

#### Mono-ubiquitination of HES1 has no effect on its cellular localization

**Fig. 2:**
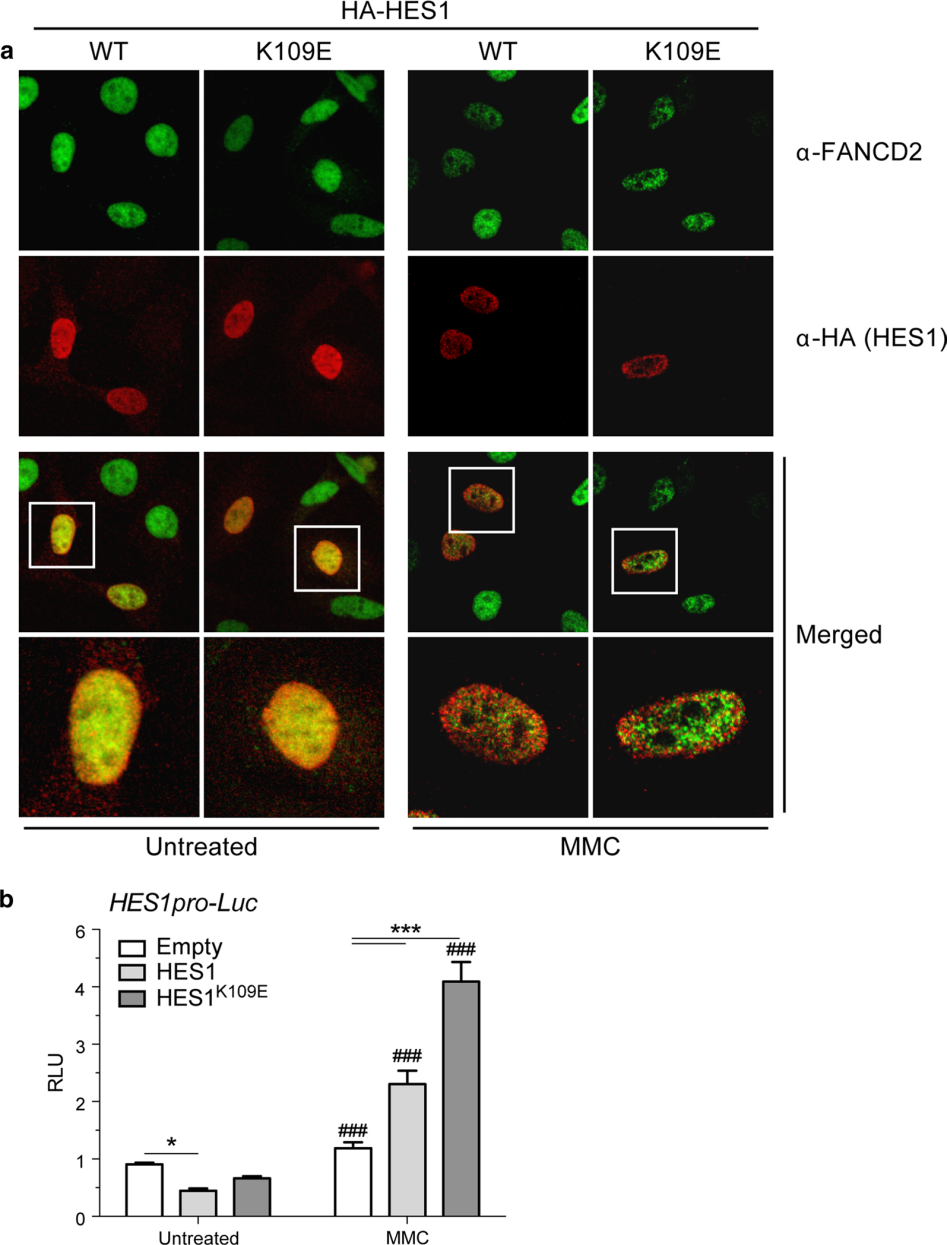
HES1 mono-ubiquitination is not required for foci formation. **a** HES1^K109E^ mutation does not impair MMC-induced HES1 foci formation. HeLa cells transfected with HA-tagged *HES1* or *HES1*^*K109E*^ coding vectors were processed for immunofluorescence 16 h following treatment with MMC (120 ng/ml). Cells were double-stained with anti-HA and anti-FANCD2 antibodies (mouse anti-HA at 1:500; rabbit anti-FANCD2, Novus Biologicals NB100-182 at 1:2000) followed with secondary antibodies (Goat anti-mouse Alexafluor-555, ThermoScientific A-32727 and goat anti-rabbit Alexafluor-488, ThermoScientific, A-11008). Cell nuclei were labelled with TO-PRO-3 Iodide stain (1:400, ThermoScientific, T3605). Cells were visualized at 100× magnification. **b** HES1 lysine 109 plays a role in MMC-induced activation of the *HES1* promoter. COS-1 cells transfected with pHES1pro-*Luc* and *HES1* or HES1^K109E^ coding vectors (0.034 μg) were treated with 120 ng/ml MMC. Empty vectors were used as controls. Experiments were done twice in triplicates. 2-way ANOVA with Bonferroni correction test, *p < 0.01 and ***p < 0.0001, as compared to Empty; ^###^p < 0.0001, as compared to Untreated

We previously showed that HES1 is partially localized to FANCD2-containing foci in MMC-treated cells [5]. Given that MMC-induced nuclear foci contain the mono-ubiquitinated form of FANCD2, we tested whether HES1 mono-ubiquitination is required for localization to MMC-induced foci. Results show that both wild-type and K109 mutant forms of HES1 are located to the nucleus (Fig. 2a, right panels). In addition, MMC treatment of HES1-transfected cells reveals partial localization of wild-type and mutant HES1 to FANCD2-conatining nuclear foci (Fig. 2a, left panels). These results indicate that mono-ubiquitination has no impact on HES1 cellular localization in non-treated and MMC-treated cells.

Given that HES1 binds its own promoter to repress its own transcription [4, 12], we tested whether HES1 post-translational modification regulates this auto-negative feedback mechanism. Using the *HES1* promoter region to drive the luciferase gene (HES1pro-*Luc*), we tested the transcriptional repression capacity of HES1 K109-mutant. Results show that HES1^K109E^ has reduced transcriptional repression capacity compared to the wild-type HES1 protein (Fig. 2b). Considering that the FA core complex ubiquitin ligase activity is known to modify its substrates in response to DNA damage [7–9], we tested whether HES1 or HES1^K109E^ transcriptional repression is modulated following DNA damage. Results show that MMC treatment induced activation of the *HES1* promoter as compared to untreated cells (Fig. 2b). Surprisingly, HES1 failed to repress its own promoter following MMC treatment as shown by a two-fold *HES1* promoter activation compared to control cells. In addition, the HES1^K109E^ further increased *HES1* promoter activation compared to control cells and untreated cells. These results suggest the MMC treatment promotes *HES1* promoter activation by preventing HES1-mediated repression. These results also suggest that the HES1 lysine 109 acts as a regulation site for its repressor function.

#### Mono-ubiquitination of HES1 is required for transcriptional repression of its own promoter

**Fig. 3:**
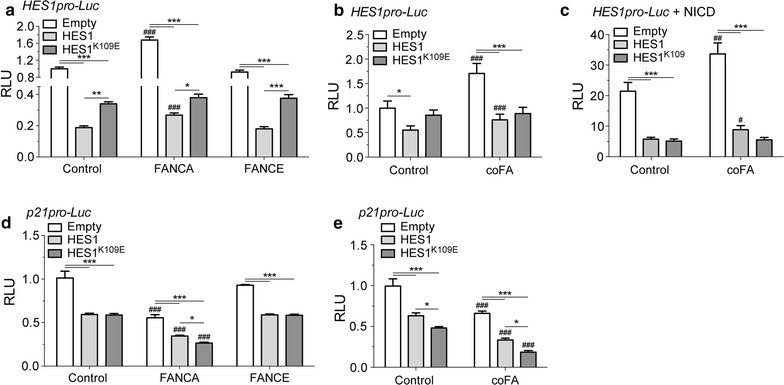
HES1 lysine 109 is required for *HES1* transcriptional regulation. **a** Altered *HES1* expression in FA mutant cells. FA-A cells were transfected with pHES1pro-*Luc* and plasmids carrying either the correcting gene *FANCA* or, as a negative control, *FANCE*; with the *HES1* or the *HES1*^*K109E*^ expression plasmid. **b** HES1 lysine 109 is required for FA complex-mediated regulation of *HES1*. COS-1 cells were transfected with pHES1pro-*Luc* and *HES1 or HES1*^*K109E*^ expression vector with or without plasmids encoding the FA core complex (coFA) and in **c** with *NICD* expression plasmid. **d** Mutation of HES1 lysine 109 promotes FA complex-mediated *p21*^*CIP1/WAF1*^ repression. COS-1 cells were transiently transfected with p21pro-*Luc* reporter vector with HES1 or HES1^K109E^ coding vectors, and either with FA complex members coding vectors. **e** FA complex-mediated repression of *p21*^*CIP1/WAF1*^ transcription is enhanced by HES1 K109E mutation. FA-A cells were transiently transfected with p21pro-*Luc* and either *FANCA* or *FANCE* (negative control) coding vectors, along with wildtype or K109E mutant *HES1* coding vectors. All plasmids were transfected at equimolar ratios. Control indicates empty vectors. All experiments were done at least three times in duplicates. 2-way ANOVA with Bonferroni correction test, *p < 0.01, **p < 0.005, *** p < 0.0001, as compared to Empty; ^#^p < 0.01, ^##^p < 0.001 and ^###^p < 0.0001, as compared to Control

To determine whether FA complex-mediated regulation of *HES1* occurs via mono-ubiquitination of HES1 lysine 109, we evaluated *HES1* gene activation in FA-A cells in the presence of HES1 or HES1^K109E^. Results show that *HES1* transcriptional regulation is impaired in FA-A cells and in *FANCE*-transfected FA-A cells but restored in FANCA-corrected cells, thus confirming previous findings (Fig. 3a). We also confirmed that HES1-mediated repression of its own promoter is attenuated in FANCA-corrected cells compared to control FA-A cells and *FANCE*-transfected FA-A cells (Fig. 3a). However, we found that HES1^K109E^ failed to repress its own promoter in FA-A cells despite FANCA-gene correction (Fig. 3a) or overexpression of FA core complex components (Fig. 3b).

Given that expression of the Notch1 intracellular domain (NICD), which is the active form of Notch1, has been shown to activate *HES* genes, including *HES1*, and that FA complex components enhanced NICD-mediated activation of the *HES1* promoter, we tested the effect of HES1^K109E^ on NICD-mediated *HES1* activation. We found that, like HES1, HES1^K109E^ repressed its own promoter independently of NICD-mediated activation of *HES1* (Fig. 3c). Surprisingly, FA core-complex components failed to enhance NICD-mediated activation of *HES1* in the presence of HES1^K109E^ (Fig. 3c). Together, these results suggest that HES1 mono-ubiquitination is required for repression of its own promoter and is required for transcription of *HES1* following NICD-mediated activation in a FA core complex dependent manner.

We next determined whether the FA core complex modulates HES1-mediated repression of *p21*^*CIP1/WAF1*^ via mono-ubiquitination of HES1. We found that HES1^K109E^ was able to repress the *p21*^*CIP1/WAF1*^ promoter more efficiently than the wild-type HES1 protein (Fig. 3d). Overexpression of FA core complex components further increased HES1^K109E^-mediated repression of the *p21*^*CIP1/WAF1*^ promoter. In addition, we found that HES1^K109E^ like wild-type HES1, repressed the *p21*^*CIP1/WAF1*^ promoter in FA-mutant cells, whereas complementation of FA-A cells with FANCA but not FANCE further increased this repression (Fig. 3e). These results suggest that mono-ubiquitination of HES1 is not required for transcriptional repression of the target gene *p21*^*CIP1/WAF1*^.

### Discussion

The HES1 protein is highly regulated through an oscillation-type mechanism, which occurs in part by an auto-negative feedback mechanism at the transcription levels and rapid ubiquitination-dependent proteosomal degradation [13, 14]. Recently the RBX1-CUL1-SKP1 ubiquitin ligase complex was shown to regulate HES1 degradation via poly-ubiquitination on several lysine residues [15]. In this publication, Chen et al. [15] revealed that HES1 poly-ubiquitination occurs at the conserved lysine residue identified as 106 in their publication but identified as 109 in NCBI reference sequence NP_005515.1. This conserved lysine residue is located within a motif similar to the FA recognition sequence found in FANCD2 and FANCI [7–9]. The fact that the same site of HES1 post-translational modification was identified using different approaches underline the possibility that this conserved residue or protein sequence motif plays a role in HES1 regulation. Indeed, our results suggest that modification of the K109 play a role in HES1 transcriptional regulation. In addition, the structural similarities between the FA core complex and the RBX1-CUL1-SKP1 complex, as proposed by Boisvert and Howlett [16], the direct interaction of HES1 with several members of the core complex [5], and our results presented here suggest that HES1 is a putative target of the FA core complex. Furthermore, we found that HES1^K109E^ lysine mutant could still interact with members of the FA the core complex (data not shown). Based on our previous findings [6] and results presented here we can hypothesize that mono-ubiquitination of HES1 influence its affinity to specific promoters and or to other corepressors.

More recently, mutations in the Ring Finger and WD Repeat Domain 3 (*RFWD3* or *FANCW*)—another E3 ligase—was shown to cause FA in a child [17]. Given the complexity of the FA pathway, now composed of two E3 ligases, it is conceivable that other substrates exist. Our results strongly suggest that HES1 is a putative substrate of the FA core complex ubiquitin ligase.

### Limitations

It is unclear whether HES1 mono-ubiquitination described in this work affects its stability and affinity to promoters or binding to corepressors. It is also conceivable that HES1 mono-ubiquitination occurs via other E3 ligases regulated by the FA core complex. Further work is needed to clarify these issues.

## Abbreviations

FA: Fanconi anemia
FA-A: Fanconi anemia group A
FANCA: Fanconi anemia A
FANCD2: Fanconi anemia D2
FANCI: Fanconi anemia I
FANCW: Fanconi anemia W
HA: hemagglutinin
HES1: Hairy Enhancer of Split 1
MMC: mitomycin C
p21^CIP1/WAF1^: cyclin-dependent kinase inhibitor 1
RFWD3: Ring Finger and WD Repeat Domain 3
RBX1-CUL1-SKP1: Ring-Box 1-Cullin 1-S Phase Kinase Associated Protein 1
